# Material Preparation Information File (MPIF): A Community‐Driven Standard for Reporting MOF Syntheses

**DOI:** 10.1002/adma.202521420

**Published:** 2026-02-15

**Authors:** Ocean Cheung, Shun Tokuda, Damian Jędrzejowski, Evelyn Ploetz, Bettina Baumgartner, Marzena Pander, Fengxu Yang, Jack D. Evans, Romy Ettlinger, Stefan Wuttke, Dariusz Matoga

**Affiliations:** ^1^ Department of Materials Science and Engineering The Ångström Laboratory Uppsala University Uppsala Sweden; ^2^ Max Planck Institute for Solid State Research Stuttgart Germany; ^3^ Academic Centre For Materials and Nanotechnology AGH University of Kraków Kraków Poland; ^4^ Faculty of Chemistry Jagiellonian University Kraków Poland; ^5^ Department of Chemistry and Center of NanoScience (CeNS) Ludwig‐Maximilians‐Universität München Munich Germany; ^6^ Van't Hoff Institute for Molecular Sciences University of Amsterdam Amsterdam the Netherlands; ^7^ School of Physics Chemistry and Earth Sciences The University of Adelaide Adelaide Australia; ^8^ TUM School of Natural Sciences Technical University of Munich Garching bei München Germany

**Keywords:** data availability, materials synthesis, metal‐organic frameworks, MPIF, open science

## Abstract

The rapid growth of research on metal–organic frameworks (MOFs) and related porous materials has highlighted a critical need for standardized reporting of synthetic procedures and material properties. Synthesis consistency is often compromised by incomplete protocols and inconsistent nomenclature, with even well‐established MOFs exhibiting substantial variability depending on subtle and frequently unreported factors. To address this critical challenge, we introduce the Material Preparation Information File (MPIF), a modular, machine‐ and human‐readable format for documenting synthesis protocols alongside key characterization data. Built on the Self‐defining Text Archive and Retrieval (STAR) file architecture, MPIF consolidates essential information, including reagents, conditions, equipment, and handling procedures—into a structured format that supports both digital processing and practical laboratory use. To streamline MPIF creation and editing, a web‐based interface has been developed (mpif.jackdevans.com). By aligning with existing standards such as CIF and AIF, MPIF enables integration with databases, promotes FAIR data principles, and provides a foundation for data‐driven materials discovery, informatics, and automation. We envision MPIF becoming a community‐wide standard for transparent and consistent reporting of MOF and related material syntheses.

## Graphical abstract

The Material Preparation Information File (MPIF) establishes a universal, community‐driven format for documenting the synthesis of metal–organic frameworks and related materials. By capturing complete, machine‐readable synthesis and characterization details, MPIF enhances reproducibility, supports FAIR data sharing, and bridges experimental chemistry with digital materials discovery and automation.

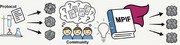

## 1 Introduction

Framework porous materials – including metal‐organic frameworks (MOFs), covalent organic frameworks (COFs), and hydrogen‐bonded organic frameworks (HOFs)–have attracted significant academic and industrial attention in recent years [1]. With over 100 000 MOF structures synthesized and reported to date [2], research in this area is progressing rapidly. These porous materials, known for their large internal surface areas and tunable pore chemistries, have found uses in a wide range of applications, including nanoscale technologies like drug delivery, biological imaging, and environmental applications such as water harvesting, water purification, air filtration, building insulation, and, more recently, carbon capture [3]. A range of carefully engineered MOFs, such as MOF‐801, MOF‐808, and MOF‐303, have been tested and applied for atmospheric water harvesting [4, 5, 6, 7]. CALF‐20 has been recently implemented in large‐scale carbon capture applications, overcoming long‐standing skepticism regarding production costs, chemical and physical stability, and application feasibility of MOFs [8].

However, the rapid growth of the MOF field has also outpaced its internal standardization. The community finds itself at a turning point: after years of publishing a large number of new structures, the urgent question now is how to ensure consistency, comparability, and reliability across laboratories and large‐scale production facilities [9, 10]. Nomenclature inconsistencies, vague experimental reporting, and heterogeneous analytical approaches have created challenges that hinder both scientific progress and industrial translation, not only for MOFs but also in other fields within materials and chemical science.

**SCHEME 1:**
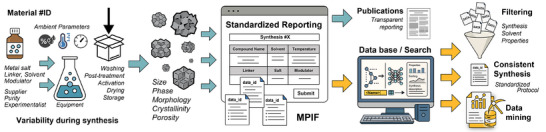
Schematic diagram illustrating the concept and objectives of this work. The Material Preparation Information File (MPIF) is a text‐based format designed to systematically collect highly detailed synthesis information. MPIF reduces the omission of critical details in synthesis reporting within text‐based literature. The blue arrow represents the work completed in this study, while the yellow arrows indicate possible immediate extensions and applications of MPIF.

It is now widely recognized that the continued advancement of MOFs depends on the establishment of robust standards for documenting and reporting synthesis and material properties, such as those highlighted by Herres‐Pawlis et al. [11]. The development of Crystallographic Information Files (CIFs) [12] and, more recently, Adsorption Information Files (AIFs) [13], as well as the guidelines on the terminology for coordination polymers and metal organic frameworks published by IUPAC [14], have all played a crucial role in advancing MOF research. Whereas CIF and AIF are explicitly designed to describe a single type of measurement or structure (crystal structures and adsorption isotherms, respectively), MPIF is aimed at end‐to‐end, machine‐readable synthesis and characterization reporting for MOFs. Related efforts such as ULSA (Unified Language of Synthesis Actions) provide a controlled vocabulary for annotating synthesis actions to support machine parsing, but ULSA is an action‐level language for semantic annotation rather than a standalone archival file format [15]. SMART Protocols and similar reporting checklists, used primarily in biological and biomedical experiments, prescribe minimal, machine‐readable protocol elements required for reproducibility but do not define a single, domain‐specific file format tailored to materials synthesis and characterization [16]. Industrial frameworks such as the Allotrope Data Format (ADF) provide rich ontologies and instrument‐centric containers optimized for analytical data provenance and large dataset packaging, yet their complexity and focus on instrumentation make them less suitable as lightweight, human‐editable synthesis and characterization records for routine MOF workflows [17]. Materials Acceleration Platforms (MAPs) [18], applied, for example, in polymer science, offer high‐throughput, closed‐loop discovery infrastructures; MPIF is complementary rather than competitive: it provides a material‐specific, machine‐readable synthesis and characterization record that MAP stacks can ingest to enhance reproducibility and downstream analytics. A standardized reporting format for the synthesis of MOFs and related materials is essential. Not only would it improve the efficiency of developing both new and existing MOFs, but it would also harmonize synthesis practices across different scales (laboratory, pilot, and industrial production) and support computer‐based advancements such as data mining, large language models (LLMs), and automated MOF production (Scheme 1).

**FIGURE 1:**
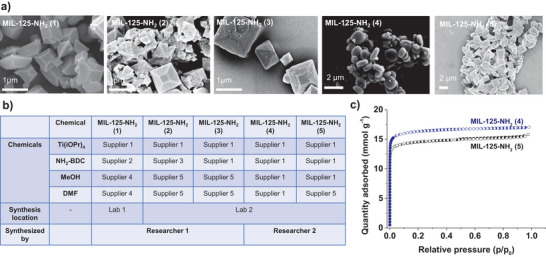
(a) Scanning electron micrographs of five different samples of MIL‐125‐NH_2_ synthesized following the same procedure under different environments with chemicals from different suppliers; (b) lab conditions and chemical suppliers under which the five MIL‐125‐NH_2_ were synthesized; (c) N_2_ adsorption/desorption of MIL‐125‐NH_2_ (4) and MIL‐125‐NH_2_ (5), samples were prepared under the same conditions after synthesis.

The synthesis of MOFs can be highly sensitive, and protocols are frequently incomplete. Even under seemingly identical synthesis conditions, materials can differ substantially due to minor, often unreported factors such as the supplier of chemicals, the type of glassware, small differences in ambient temperature, or humidity during synthesis. As a result, even well‐established MOFs such as MIL‐125‐NH_2_ can exhibit substantial variability. Figure 1a shows the SEM images of MIL‐125‐NH_2_ (1)‐(5) samples synthesized using chemicals from different suppliers as well as at two different laboratories following an identical experimental procedure. Even within one laboratory, differences in MOF synthesized with the same procedure can also arise; this could be demonstrated by comparing the data shown in Figure 1a for MIL‐125‐NH_2_ (4) and MIL‐125‐NH_2_ (5) and the N_2_ sorption isotherms of these samples as shown in Figure 1c. These two MIL‐125‐NH_2_ samples were made by one researcher in the same lab following an identical procedure, with the only difference being the supplier of the organic solvent used in the synthesis. These results highlight the extent of variability that can arise even under controlled conditions and underscore a broader issue: the lack of standardized protocols for reporting MOF synthesis and characterization. As a result, critical details are frequently omitted from published reports. Standard operating procedures (SOPs) can vary significantly between laboratories. The variation means that even when technically, the same synthesis procedures were adopted, the MOFs produced by different labs may not be directly comparable. In many instances, the reported synthesis protocols are optimized for specific laboratory conditions; deviating from these conditions can substantially affect the physical properties of the MOF. In extreme cases, MOFs described in the literature may prove irreproducible by other researchers or laboratories, with successful synthesis limited solely to the original reporting group.

The consequence of missing standards is a proliferation of synthesis procedures for nominally identical MOFs, with substantial differences in structural and functional properties. Moreover, these procedures often differ markedly in terms of reaction conditions and reagent compositions. As a result, different MOF samples that vary significantly in crystallinity, morphology, phase, and physical properties are frequently labelled as the same MOF. The opposite can also happen, where two isostructural MOFs could independently be assigned different names by different researchers. The differences in the properties between different MOFs with the same structure can often be rationalized, but they are rarely emphasized in the literature, further complicating the consistency in the literature as well as the possibility for comparative analysis.

Moreover, the absence of a standardized reporting format presents a significant barrier to the uptake of modern data science approaches. For example, the ability to systematically data‐mine experimental conditions using tools like LLM has shown promise in MOF research, where diverse synthesis protocols, adsorption measurements, and structural descriptions are scattered across thousands of publications. For example, a ChatGPT‐based extraction agent developed by Zheng et al. achieved precision and recall scores over 90% for capturing synthesis parameters such as volumes and reaction temperature from the literature [19]. Similarly, MOFh6 LLM framework demonstrated a stable precision of 0.93 ± 0.01 for extracting various chemicals and experimental conditions [20]. However, to improve and support the use of artificial intelligence assistants in this area, structured, consistent, and machine‐readable protocols are necessary. This framework would enable researchers to uncover subtle trends in synthesis conditions, predict outcomes, and rapidly identify the most effective procedures for producing a material with desired properties for a given application, thereby accelerating materials discovery and optimization.

Standardizing the reporting of MOF synthesis procedures and associated material properties is essential to addressing the challenges in synthesis and product consistency, as well as future data mining efforts, as outlined above. For such standards to be effective, reports must be presented in one specific format and include comprehensive details of the synthesis process. Additionally, basic characterization data of the final product should accompany the synthesis description, enabling researchers to identify the MOF structure and compare their own results with the original benchmarks. To this end, we propose the Material Preparation Information File (MPIF) format, designed to facilitate standardized and accurate reporting of MOF synthesis protocols alongside relevant characterization data. A major advantage of the MPIF format is that it supports both modern data processing and practical use by chemists. Built on the STAR (Self‐defining Text Archive and Retrieval) structure, MPIF organizes synthesis information using clearly labeled fields and repeatable data blocks, making it easy to capture and interpret all relevant details—such as chemical identities, reaction conditions, equipment used, and lab environment. For chemists, this means that MPIF offers a clear and complete framework for documenting synthesis procedures without overlooking critical details.

We have also developed a web‐based interface [21] (accessible through the MPIF information page www.mpif4ever.com, or directly at mpif.jackdevans.com) that enables users to generate MPIF files through simple input forms. This platform supports both the creation and visualization of MPIF files, allowing researchers to review and share detailed synthesis records efficiently. We envision MPIF becoming a standard supporting information file for future publications involving materials synthesis, in particular for MOFs and COFs, but also for other materials such as zeolites. As machine‐readable records, MPIF files will also pave the way for large‐scale data mining and informatics‐driven materials discovery. As ongoing work to further develop and promote community‐wide adoption of MPIF, we are in discussion with journal editors at different publishers. We have also responded to early feedback from the community and have begun working on incorporating MPIF into electronic laboratory notebooks (ELNs) as well as major databases. The mechanism and impact of MPIF are illustrated in Scheme 1.

## 2 Results and Discussion

### 2.1 MPIF Data Structure

The MPIF was designed to consolidate synthesis and characterization details of a material within a single, machine‐ and human‐readable file. The STAR data structure that MPIF adopts is the same as that used in CIF, AIF, and nuclear magnetic resonance spectroscopy (NMR)‐STAR. STAR organizes information through “data items”, which are pairs of names and corresponding values, and supports both single entries and tabular data via the “loop_” construct. This flexible structure accommodates metadata (e.g., author information), repetitive elements (e.g., details of multiple substrates), and series data (e.g., data points from PXRD profiles).

Each MPIF file corresponds to one material preparation and therefore contains only one data block, whose beginning row is uniquely identified by a “data_X” statement. Within this block, data are stored either as individual _name value pairs or in tables defined by loop_. Comment lines prefixed by # may be added, but are discouraged for essential information. This design ensures that the MPIF format remains both compact and extensible.

**FIGURE 2:**
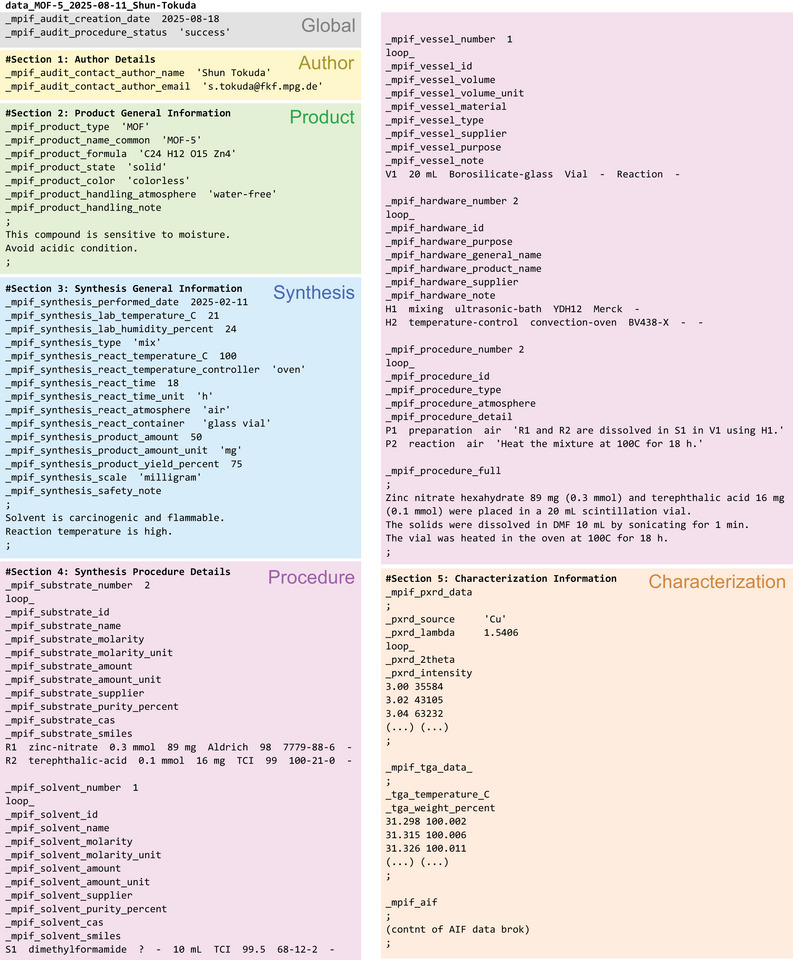
Typical data structure of MPIF. Each background color represents a different section.

The content of one MPIF data block is divided into two main parts: synthesis procedure information and characterization information. A typical file structure of an MPIF is shown in Figure 2 and contains:

Section 1: Global Metadata of the file (file creation date, associated publication Digital Object Identifier (DOI), etc.).

Section 2: Author Details specifies basic information about the person responsible for preparing the MPIF. Product General Information defines details about the material to be prepared, such as name, composition, appearance, and handling conditions. It is possible to embed the CIF materials in this section.

Section 3: Synthesis General Information presents general information about the synthesis process and key parameters (e.g., reaction temperature). Essential data, such as laboratory temperature and humidity, are included here, while Table S1 clearly specifies which of these items are mandatory and which are optional.

Section 4: Synthesis Procedure Details is the core of the MPIF, which has a structure composed of five sub‐sections that specify details about the Reactants (= substrates, metal, linker, modulator, etc.), Solvents, Vessels, Hardware, and Procedure. Each of these five categories has a tabular structure; the author specifies the number of entries in each category and then defines details for each item, such as quantity, purity, purpose of use, or a simplified description of the operation. Modulators are treated as substrates, reflecting their ambiguous role in MOF synthesis.

Section 5: Characterization Information, which serves to integrate synthesis protocols with a minimum set of characterization data collected on the prepared material. At present, authors can include raw data from a powder X‐ray diffraction (PXRD), thermogravimetric analysis (TGA), as well as the full content of AIF directly within the MPIF file. All characterization data are stored as ASCII text, typically formatted as simple two‐column data tables. The current implementation is intentionally conservative and text‐based, but not restrictive. Future updates of the GUI are expected to accommodate additional data representation, including structured JSON formats, as well as direct references to external repositories such as Zenodo or Figshare. Depending on the application and data complexity, more advanced container formats–such as HDF5–may be considered to enable the inclusion of full measurement datasets or multi‐dimensional data acquired during characterization. Importantly, the implementation of these characterization techniques in the MPIF format is not exclusive. Encoding measurement data from other techniques in a STAR‐compliant format will allow for the future expansion to include other characterization methods, such as infrared (IR) or NMR spectrum data.

### 2.2 MPIF Data Items

While the last Section 2.1 describes the file's overall organization, this section details the data item nomenclature and conventions used within an MPIF. All MPIF data names begin with a common prefix _mpif, e.g., _mpif_synthesis_lab_temperature, unlike CIF and AIF, intended to avoid any conflict of data names with other STAR format files. Data items grouped by families are indicated in the second part of the names (e.g., _audit, _product, _synthesis, _substrate, _solvent, _vessel, _hardware, _procedure, etc.). A curated list of the principal MPIF data names is presented and explained in Table 1, whereas a comprehensive dictionary of accepted data names can be found in Table S1.

**TABLE 1:** Core data items in MPIF. See the Supporting Information for the full list of data items.

Data name	Description (Data type)
Global metadata	
_mpif_audit_publication_doi	DOI of the associated publication (string)
_mpif_audit_procedure_status	classification of the procedure (string from {test, successful, failed})
Section 1: Author details	
_mpif_audit_contact_author_name	name of the responsible author (string)
Section 2: Product general information	
_mpif_product_name_common	common name of the product (string)
_mpif_product_state	physical state of the product (string from {solid, liquid, gas, suspension, other})
_mpif_product_color	color of the product (string)
_mpif_product_handling_atmosphere	recommended atmosphere for handling the product (string from {air, inert, water‐free, oxygen‐free, other})
_mpif_product_handling_note	special note for handling the product (string)
_mpif_product_cif	embedded CIF (string)
Section 3: Synthesis general information	
_mpif_synthesis_performed_date	date of the experiment (ISO 8601 format)
_mpif_synthesis_lab_temperature_C	room temperature in Celsius (float)
_mpif_synthesis_lab_humidity_percent	room relative humidity in percent (float)
_mpif_synthesis_type	type of the synthetic procedure (string from {mix, diffusion, evaporation, microwave, mechanochemical, electrochemical, sonochemical, photochemical, flow, other})
_mpif_synthesis_react_temperature_C	reaction temperature in Celsius (float)
_mpif_synthesis_react_temperature_controller	method of controlling reaction temperature (string from {ambient, oven, oil bath, water bath, dry bath, hot plate, microwave, furnace, other})
_mpif_synthesis_react_time	reaction time (float)
_mpif_synthesis_react_atmosphere	reaction atmosphere (string from {air, dry, inert, vacuum, other})
_mpif_synthesis_react_container	reaction container (string)
_mpif_synthesis_react_note	special note for reaction (string)
_mpif_synthesis_product_amount	amount of the product (float)
_mpif_synthesis_product_yield_percent	product yield in percent (float)
_mpif_synthesis_scale	scale of the synthesis (string from {milligram, gram, multigram, kilogram})
_mpif_synthesis_safety_note	safety note for the synthesis (string)
Section 4: Synthesis procedure details	
_mpif_substrate_id	identification code of the substrate (string: from {R1, R2, …})
_mpif_substrate_name	name of the substrate (string)
_mpif_substrate_molarity	molar amount of the substrate (float)
_mpif_substrate_amount	mass or volume of the substrate (float)
_mpif_substrate_supplier	supplier of the substrate (string)
_mpif_substrate_purity_percent	purity of the substrate in percent (float)
_mpif_substrate_cas	CAS registry number of the substrate (string)
_mpif_substrate_smiles	SMILES of the substrate chemical structure (string)
_mpif_solvent_id	identification code of the solvent (string from {S1, S2, …})
(_mpif_solvent_*)	(_mpif_solvent has the same data items as _mpif_substrate)
_mpif_vessel_id	identification code of the vessel (string from {V1, V2, …})
_mpif_vessel_volume	volume of the vessel (float)
_mpif_vessel_material	material of the vessel (string)
_mpif_vessel_type	type of the vessel (string from {vial, jar, autoclave, beaker, flask, centrifugation tube, other})
_mpif_vessel_supplier	supplier of the vessel (string)
_mpif_vessel_purpose	purpose of the vessel (string from {storing, reaction, other})
_mpif_vessel_note	special note of the vessel (string)
_mpif_hardware_id	identification code of the hardware (string from {H1, H2, …})
_mpif_hardware_purpose	purpose of the hardware (string from {temperature control, atmosphere control, mixing, synthesis devise, transferring, separation, drying, other})
_mpif_hardware_product_name	product name of the hardware (string)
_mpif_hardware_supplier	supplier of the hardware (string)
_mpif_hardware_note	special note of the hardware (string)
_mpif_procedure_id	identification code of the procedure (string from {P1, P2, …})
_mpif_procedure_type	type of the procedure (string from {preparation, reaction, work‐up})
_mpif_procedure_atmosphere	atmosphere of the procedure (string from {air, dry, inert, vacuum, other})
_mpif_procedure_detail	detailed note of the procedure (string)
_mpif_procedure_full	full details of the procedures (string)
Section 5: Characterization information	
_mpif_pxrd_data	embedded PXRD data (string)
_mpif_tga_data	embedded TGA data (string)
_mpif_aif	embedded AIF (string)

Audit items record essential metadata such as the responsible author and file creation date, inherited in part from CIF and AIF. One characteristic addition is “_mpif_audit_procedure_status”, which is given to classify the procedure as “successful” or “failed”, otherwise “test” is also accepted as a value. This metadata will be useful when one compares different procedures for one material, and more importantly, when one makes a database of MOF synthesis, including “failed” experiments.

Product items, _mpif_product, describe the resulting material, including appearance (color and physical state), and expected handling condition (e.g., inert atmosphere). Optional registry numbers, such as Chemical Abstracts Service (CAS) and Cambridge Crystal Database Centre (CCDC) identifiers, can be included to link the entry to existing references.

Synthesis items (_mpif_synthesis) capture environmental and operational details that are often underreported, such as laboratory temperature (_mpif_synthesis_lab_temperature_C), humidity (_mpif_synthesis_lab_humidity_percent), as well as atmosphere, reaction type, and safety notes. Authors must also specify the synthesis type, chosen from nine predefined options (mix, diffusion, evaporation, microwave, mechanochemical, electrochemical, sonochemical, photochemical, flow chemistry), with additional fields requested depending on the selection (see the Supporting Information for the details). Note that safety notes (_safety_note) can be provided as a long text in this section, but this is optional (as indicated in Table S1).

Section 4 is represented by five tabular families of data: Substrates, Solvents, Vessels, Hardware, and Procedure steps. Substrate entries (_mpif_substrate) are indexed (R1, R2, …), and include name, molarity, amount, name of supplier, purity, and CAS number fields. The SMILES notation (_smiles) can be used to describe complicated organic molecules. The same structure applies to Solvents (_mpif_solvent), identified by S1, S2, and so on. Vessels (_mpif_vessel) require an identifier (V1, V2, …), volume, material, and type (chosen from vial, jar, autoclave, beaker, flask, centrifugation tube, and other). Optional fields include _supplier, _purpose (selected from storing, reaction, or other), and _note. Hardware items (_mpif_hardware), similarly require an identifier_id (H1, H2, …), _general_name, and _purpose, while _product_name, _supplier, and _note are optional.

The last table field is the Procedure steps (_mpif_procedure). Each entry of the table corresponds to each step in the procedure, and is identified with P1, P2, …, and each procedure step must include three structured elements: (i) the step type (_type: preparation, reaction or work‐up), (ii) reaction atmosphere (air, dry, inert, vacuum, or other), and (iii) references to the relevant R/S/V/H codes in the step _detail (e.g., “R1 was dissolved in S2 in V1 using H3”), These minimum requirements are marked as mandatory in Table S1. The final data item of Section 4, _mpif_procedure_full, which appears outside the above loops, remains available as a human‐readable narrative analogous to an experimental section; however, it is explicitly defined as optional and should not be used as the sole source of critical procedural information. Although this information is redundant to the previous procedure table, one can effectively use this long‐text form to supplement the information that cannot be formatted into the above tables. The final section of an MPIF, Section 5, includes the physicochemical characterization data of the product material. For each characterization dataset, all numerical data and the measurement metadata are included as a long‐text value for a data name. For example, the PXRD dataset has only one data name (_mpif_pxrd_data) and only one long‐text value, in which the metadata (_pxrd_source, _pxrd_lambda) and the numerical data (_pxrd_2theta, _pxrd_intensity) are written in a STAR format. Similarly, TGA data is included in one data item (_mpif_tga_data). It should be noted that this “embedded” STAR format text is only recognized as a simple long text rather than a part of STAR syntax. Such nesting of syntax was adopted as it allows embedding AIF directly in an MPIF under the corresponding data item (_mpif_aif) without risking a conflict of data names with each other.

As discussed earlier, the STAR format allows future versions to include more detailed parameters that are not commonly archived, such as information about crystal size, reaction vessel fill ratio, or material defectivity. Proper preparation of the data format from another characterization technique may also enable its integration with the MPIF file.

### 2.3 MPIF Graphical User Interface

**FIGURE 3:**
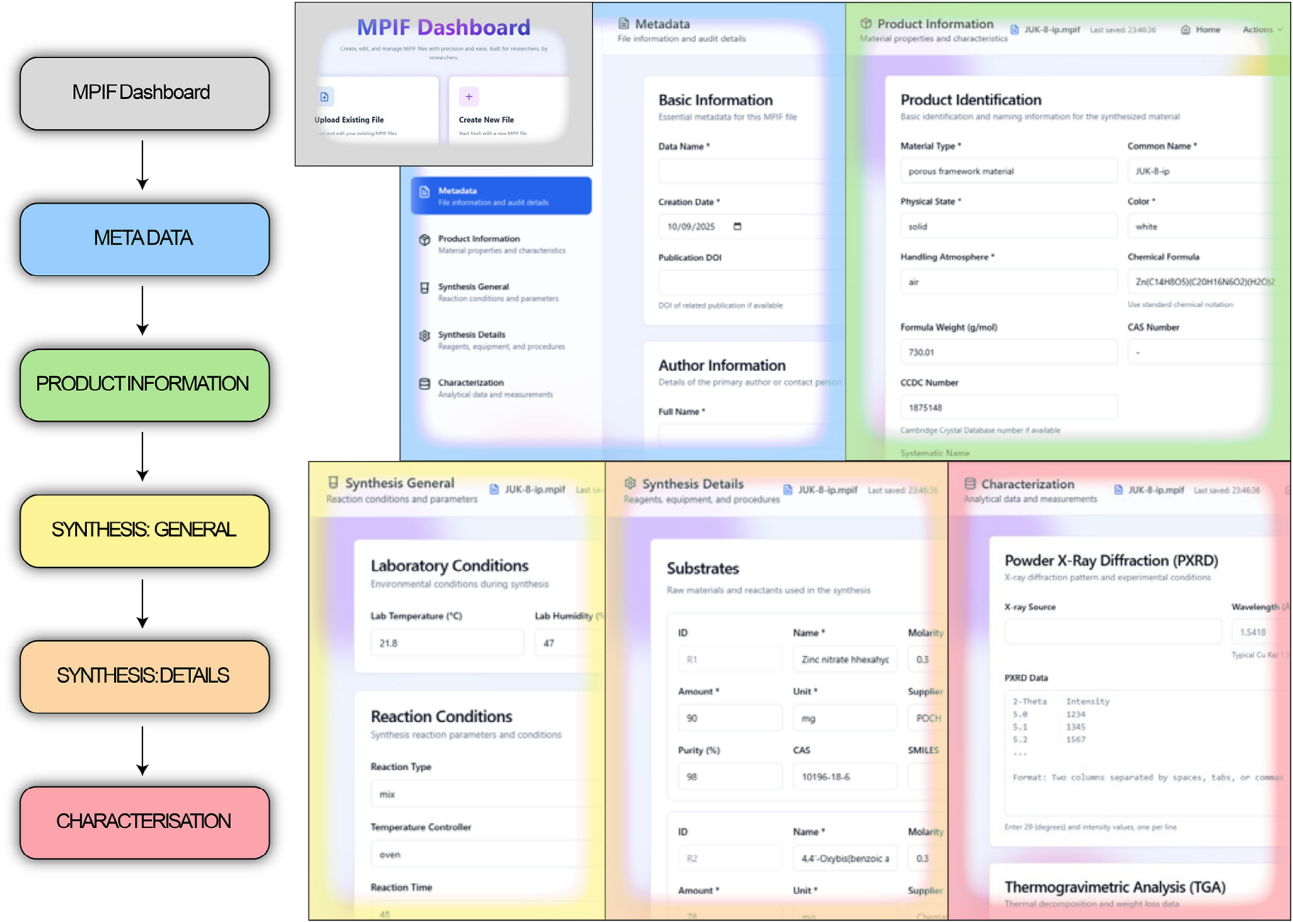
Views of the current version of the web‐based graphic user interface (GUI) of the MPIF editor with the different sections.

As CIFs and AIFs are usually prepared via dedicated graphical user interface (GUI) programs, MPIF should also be generated and edited via a GUI to prevent syntax errors and fulfill information requirements. Thus, we developed the online GUI (Figure 3) that allows for facile generation, edition, and visualization of MPIFs. The GUI is composed of several pages corresponding to each section of MPIF, where users can create a new MPIF. The interface allows the author to fill in the required information by selecting it from the drop‐down menu or as free text in text boxes, depending on the page. The different pages are divided according to those listed earlier, in Section 2.1. After filling in all required information on all pages of the GUI, one can create and export the MPIF based on the input data as a new file. All fields designated as mandatory in Table S1 are indicated in the GUI with an asterisk, and the GUI performs export‐time validation: generation of a valid.mpif file requires completion of all required fields (users are prompted to resolve missing items before export). Naturally, the GUI allows users to open and update previously created MPIFs by choosing the “Upload file” option at the top of the page. The source code generated and a record of the development efforts are publicly available on GitHub [22]. We are continually developing this GUI; for example, we aim to introduce the ability to visualise characterisation data embedded in the MPIFs in the near future.

### 2.4 MPIFs in Practice: Case Studies With Representative MOFs

**FIGURE 4:**
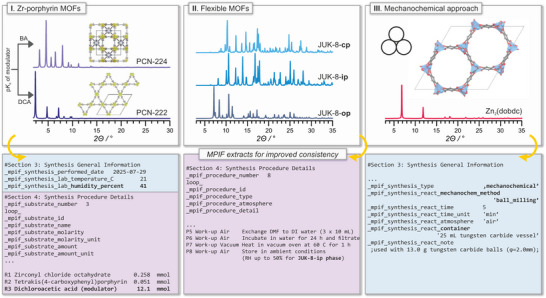
Representative classes of MOFs illustrating common challenges in synthetic consistency.

To demonstrate the applicability of MPIFs, we selected two representative MOFs that exemplify common challenges in synthetic consistency (Figure 4). These examples illustrate how MPIF facilitates the systematic documentation of critical synthesis details, ensuring that essential parameters are not overlooked, and thereby increasing the consistency across laboratories (see Supporting Information for full MPIFs). Among these, porphyrin‐based Zr‐MOFs represent a class of materials with particularly well‐documented issues in synthetic consistency [23]. For instance, the source of ZrCl_4_ and the water content strongly affect product formation [24]. Moreover, systematic analysis of published protocols has revealed that other parameters, including the pK_a_ of a modulator, the type of Zr precursor, and reagent stoichiometry, can affect nucleation kinetics and direct the formation of different crystalline phases [25]. Importantly, all of these variables are explicitly accounted for in the MPIF format, ensuring consistent reporting and enabling researchers to identify potential sources of inconsistency. To illustrate this, we synthesized PCN‐222 following a modified version of the procedure reported by Shaikh et al. [26], using ZrOCl_2_∙8H_2_O as the Zr source. The complete protocol is included in the corresponding MPIF, together with standard characterization data (PXRD, TGA, and N_2_ sorption at 77 K), which confirm the phase purity of the resulting material.

As a second example, we considered flexible MOFs, where the challenge in obtaining consistent products often arises not only from the synthesis itself, but also from post‐synthetic handling and storage. To highlight this, we selected the Zn‐based JUK‐8, a water‐stable 8‐fold interpenetrated MOF known for its pronounced breathing behavior [27]. In such cases, the product of the synthesis depends not only on precise synthetic control but also on the careful documentation of workup and storage conditions, as these directly influence which crystalline phase is observed. MPIF is a valuable tool for recording such details alongside bulk characterization data, thereby enabling unambiguous phase identification. Here, JUK‐8 was synthesized according to the reported procedure [28], and an MPIF was prepared that includes both the synthetic route and the handling steps required to obtain a distinct intermediate phase (JUK‐8‐ip), which corresponds to a hydrated phase. This semi‐stable form can be stored under ambient conditions (with relative humidity up to 50%) for extended periods and serves as a reliable indicator of successful synthesis, unlike the solvated open‐pore (op) or closed‐pore (cp) phases, which are more difficult to capture under routine measurement conditions.

While in these two examples the key factors influencing their consistency were primarily chemical—such as precursor identity, modulator choice, stoichiometry, and post‐synthetic handling—MPIF also ensures the systematic reporting of operational parameters, including reaction vessel type, reaction volume, and stirring speed. These details are often omitted from conventional experimental descriptions, yet they can significantly impact crystallization pathways and the final product quality in many MOF syntheses. Importantly, this standardized approach is not limited to solvothermal methods: MPIF can also be applied to fundamentally different synthetic strategies, such as mechanochemical synthesis. To illustrate this versatility, a representative MPIF describing the mechanochemical synthesis of Mg_2_(dobdc) was prepared based on the literature data [29]. In contrast to solvothermal examples, the corresponding MPIF explicitly records parameters that are critical for mechanochemical reactions, including the type and material of the milling jar and balls, the type of instrument used, as well as the milling time and frequency. In addition, the use of liquid‐assisted grinding (LAG), including the nature and amount of solvent, and the presence of a deprotonating agent (N,N‐diisopropylethylamine, DIPEA in this case), is unambiguously documented. By capturing this level of detail, MPIF helps minimize hidden inconsistencies and supports more effective knowledge transfer across laboratories.

### 2.5 Implication for Data Processing

**FIGURE 5:**
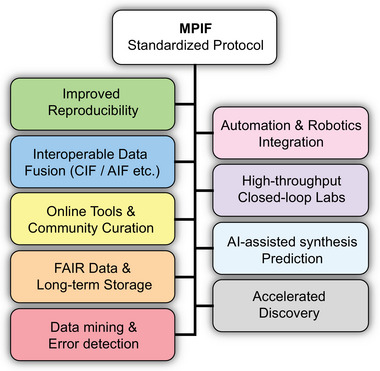
Digital transformation in MOF Synthesis: Opportunities enabled by MPIF.

The introduction of MPIF opens new perspectives for data handling and reuse in the MOF community (Figure 5) and meets the Findable, Accessible, Interoperable, and Reusable (FAIR) data principles [30]. With its modular and machine‐readable format, MPIF has several implications for how synthesis data can be processed, compared, and interpreted across laboratories, platforms, and applications. The structured format ensures that as many synthesis details as possible are recorded. This includes not only reaction parameters but also auxiliary conditions, including lab temperature and humidity, which are often essential but omitted in synthesis reporting in the literature. Furthermore, MPIF allows information regarding specific equipment (model number, make) to be included systematically in a simple manner, which can help streamline procedures for scaled‐up synthesis. To the best of our knowledge, there is currently no widely adopted, systematic way to report these details within the MOF field. This allows for better traceability and comparability across different laboratories.

In practical FAIR data workflows, this design allows MPIF to function as a synthesis‐ and characterization‐focused metadata layer that is deposited alongside raw and processed datasets in established FAIR repositories. For example, MPIF files describing the preparation history of a MOF can be uploaded together with experimental or computational data to platforms such as NOMAD or the Materials Project, where persistent identifiers (e.g., DOIs) enable direct linking between synthesis protocols, characterization results, and derived structure–property data. In this way, MPIF complements existing FAIR infrastructures by providing a standardized, machine‐readable synthesis context without duplicating or replacing repository‐specific data models.

The structure of the MPIF aligns with other existing files, including CIF for structural data and AIF for sorption properties. This alignment enables a holistic digital fingerprint of a material. In this work, we have developed a practical online tool to automatically generate, convert, and visualize MPIF files. Further developments could be the extraction of data from legacy publications or unstructured lab reports. These tools could use AI‐driven text mining or natural language processing (NLP) to extract synthesis data and convert them into MPIF format and archive them in institutional repositories, open‐access databases, or electronic lab notebooks. This structured approach supports long‐term data preservation and auditability, which are important for both academic and industrial use.

Recent efforts in other areas of material science, such as structured data representations developed for polymer systems (e.g., SPD/MAP) [18], demonstrate the growing recognition of the need for standardized, machine‐readable material data. These approaches primarily focus on encoding polymer structures, compositions, and property relationships. In contrast, MPIF addresses a complementary challenge by targeting the detailed and systematic documentation of synthesis procedures, environmental conditions during synthesis, and operational parameters that critically influence reproducibility and application in MOFs and other related materials. Rather than replacing existing data standards, MPIF is designed to integrate with them, providing a synthesis‐centric layer that connects experimental workflows with the structure‐ and property‐focused databases.

Having structured, comparable, and well‐documented synthesis records of materials can also enable further automation in synthesis and development. MPIF allows for large‐scale data mining, clustering of similar protocols, and identification of correlations between synthesis conditions and material properties. Importantly, machine learning (ML) models can now be trained not only on final MOF structures but also on the full preparation history, which offers a more realistic and predictive synthesis–structure–property relationships. To demonstrate the challenge of extracting this information from unstructured text, we tested the ability of several state‐of‐the‐art LLMs to extract key synthesis parameters from the manuscripts and Supporting Information for three different materials (full details are presented in the Supporting Information, supporting data and code are publicly available on Zenodo: https://doi.org/10.5281/zenodo.17103014). This test showed that manuscripts are a poor storage medium for this data; for instance, the synthesis reaction time (_mpif_synthesis_react_time) and product yield (_mpif_synthesis_product_yield_percent) were not correctly identified by any LLM for any material. Other parameters were also frequently missed, with correct identification rates (averaged for all materials and LLMs) of only 41.67% for the reaction time unit, 33.33% for the reaction temperature, and 25.0% for the synthesis type. This highlights the unreliability of using LLMs on unstructured text. In contrast to the immense cost of using LLMs, a simple Python function using the Gemmi library [31] can accurately extract these and all metadata from an MPIF.

Finally, MPIF can be adopted to facilitate the detection of experimental procedure deviations and errors, and can enhance quality control over materials syntheses. By recording all relevant synthesis parameters, deviations between expected and observed outcomes can be systematically traced to specific variables, such as solvent grade, stirring speed, or ambient humidity. This helps to validate protocols and prevents misinterpretation caused by hidden procedural differences. Combined with retrospective data extraction from published protocols, MPIF can also uncover gaps in the consistency in the synthesis and products, highlight outlier behaviors, and bring clarity to decades of accumulated synthesis knowledge.

## 3 Conclusion

In this work, we introduced the Materials Preparation Information File (MPIF) as a standardized, modular, and machine‐readable format for documenting MOF synthesis protocols and beyond. MPIF is a standardized, text‐based, platform‐independent file that allows materials synthesis, including essential synthesis data and parameters that are often omitted, to be presented in a systematic, commonly understandable way. MPIF is developed together with an online interface that allows for easy input, generation, and sharing of synthesis records by researchers. We provide a simple, accessible, and extensible tool that facilitates synthesis consistency, enables data‐driven materials discovery, and supports transparent scientific communication across disciplines. MPIF is compatible with existing chemical identifiers, CIF and AIF, which allows future integration with databases such as PubChem [32] or the Open Reaction Database [33]. A detailed study involving multiple laboratories, highlighting improvements in synthesis consistency when using MPIF, is underway.

The first release of MPIF lays the groundwork; further development will be shaped by the needs and contributions of the broader community. We foresee future versions including advanced measurement data, and potentially integrating hybrid formats—for instance, combining the human‐readable STAR format with hierarchical HDF5 structures to accommodate larger, multi‐dimensional datasets (e.g., operando measurements, in situ microscopy, or multi‐modal spectroscopy). MPIF will pave the way for automated data mining and ML approaches to extract synthesis–structure–property relationships.

Our current focus is to have all major publishers encourage—and ultimately require—MPIF upon submission of relevant manuscripts in the near future, as well as to incorporate MPIF into popular ELNs such as LabArchives. We invite the community to contribute toward these goals. We would also be interested in establishing MPIF as a major data source for data mining, with the help of the community. Full‐scale adoption of MPIF will only be possible with community support, and we strongly encourage readers to help promote MPIF by including it in their next publication. To support long‐term adoption and sustainability of MPIF, collaborations with major research institutions and standardization bodies such as Karlsruhe Institute of Technology (KIT), International Union of Pure and Applied Chemistry (IUPAC) [34], FAIRmat [35], or the CSD‐MOF initiative [36] will be essential. These partnerships would help align MPIF with emerging international frameworks for digital chemistry and materials informatics, and ensure compatibility with future formats like OPTIMADE or FAIR‐compliant data platforms. MPIF provides a practical, inclusive bridge between traditional experimental practice and emerging digital frameworks. MPIF provides a platform for facilitating data transparency and strengthening open science. We encourage that MPIFs be required and included as Supporting Information of research publications that deal with the synthesis of materials, in particular framework porous materials such as MOFs and COFs.

## Funding

European Union (European Cooperation in Science and Technology) For the COST Action – EU4MOFs: European metal–Organic Framework network: Combining Research and Development to Promote Technological Solutions (CA22147), Swedish Research Council (Grant Nos. 2020–04029 and 2025‐04451), Olle Engkvist Foundation, Australian Research Council Discovery Early Career Award (project number DE220100163), Fonds der Chemischen Industrie, National Science Centre, Poland (project numbers: 2024/55/B/ST5/01448, 2025/57/B/ST5/04899).

## Conflicts of Interest

The authors declare no conflicts of interest.

## Supporting information




**Supporting File 1**: adma72556‐sup‐0001‐SuppMat.docx.

**Supporting File 2**: adma72556‐sup‐0002‐DataFile.txt.

**Supporting File 3**: adma72556‐sup‐0003‐DataFile.txt.

**Supporting File 4**: adma72556‐sup‐0004‐DataFile.txt.

## Data Availability

Full list of MPIF core data items, additional details for LLM data extraction (code is publicly available on Zenodo: https://doi.org/10.5281/zenodo.17103014), example MPIFs are available as supporting information. Source code for the MPIF GUI is available at https://github.com/fengxuyy/MSIF‐GUI.
