# Supplementary material for: Material Preparation Information File (MPIF): A Community‐Driven Standard for Reporting MOF Syntheses
Source: Adv Mater. 2026 Feb 15;38(16):e21420. doi: 10.1002/adma.202521420 (PMC12994321; doi:10.1002/adma.202521420)
Supplement: Supplementary file 1 — Supporting File 1: adma72556‐sup‐0001‐SuppMat.docx. [file ADMA-38-e21420-s004.docx]

Supporting Information

Material Preparation Information File (MPIF): A Community-Driven Standard for Reporting MOF Syntheses

Ocean Cheung,* Shun Tokuda, Damian Jędrzejowski, Evelyn Ploetz, Bettina Baumgartner, Marzena Pander, Fengxu Yang, Jack D. Evans, Romy Ettlinger, Stefan Wuttke, and Dariusz Matoga*

1. **MPIF data format**

**Table S1. Full list of data items used in MPIF format**

| data name | description (data type) |
| --- | --- |
| Global metadata of the MPIF | |
| _mpif_audit_creation_date | date of the file creation (ISO 8601 format), **mandatory** |
| _mpif_audit_generator_version | version of MPIF generator (string), optional |
| _mpif_audit_publication_doi | DOI of the associated publication (string), optional |
| _mpif_audit_procedure_status | classification of the procedure (string from {test, successful, failed}), **mandatory** |
| Section 1: Author details | |
| _mpif_audit_contact_author_name | name of the responsible author (string), **mandatory** |
| _mpif_audit_contact_author_email | email address of the responsible author (string), **mandatory** |
| _mpif_audit_contact_author_id_orcid | ORCID of the responsible author (string), **mandatory** |
| _mpif_audit_contact_author_address | business address of the responsible author (string), optional |
| _mpif_audit_contact_author_phone | phone number of the responsible author (string), optional |
| Section 2: Product General Information | |
| _mpif_product_type | type of product (string from {MOF, COF, composite, other}), **mandatory** |
| _mpif_product_cas | CAS registry number of the product (string), optional |
| _mpif_product_ccdc | CCDC repository number of the product (float), optional |
| _mpif_product_name_common | common name of the product (string), **mandatory** |
| _mpif_product_name_systematic | systematic name of the product (string), optional |
| _mpif_product_formula | chemical formula of the product (string), optional |
| _mpif_product_formula_weight | formula weight of the product (float), optional |
| _mpif_product_state | physical state of the product (string from {solid, liquid, gas, suspension, other}), **mandatory** |
| _mpif_product_color | color of the product (string), **mandatory** |
| _mpif_product_handling_atmosphere | recommended atmosphere for handling the product (string from {air, inert, water-free, oxygen-free, other}), **mandatory** |
| _mpif_product_handling_note | special note for handling the product (string), optional |
| _mpif_product_cif | embedded CIF (string), optional |
| Section 3: Synthesis General Information | |
| _mpif_synthesis_performed_date | date of the experiment (ISO 8601 format), optional |
| _mpif_synthesis_lab_temperature_c | room temperature in Celsius (float), optional |
| _mpif_synthesis_lab_humidity_percent | room relative humidity in percent (float), optional |
| _mpif_synthesis_type | type of the synthetic procedure (string from {mix, diffusion, evaporation, microwave, mechanochemical, electrochemical, sonochemical, photochemical, flow, other}), optional |
| _mpif_synthesis_react_evap_method | string from {ambient, reduced pressure, spray drying, other}, optional |
| _mpif_synthesis_react_microwave_power_W | microwave power in Watt (float), optional |
| _mpif_synthesis_react_mechanochem_method | mechanochemical method (string from {ball milling, grinding, screw extruder, other}), optional |
| _mpif_synthesis_react_electrochem_cathod | cathode material (string), optional |
| _mpif_synthesis_react_electrochem_anode | anode material (string), optional |
| _mpif_synthesis_react_electrochem_reference | reference electrode (string), optional |
| _mpif_synthesis_react_electrochem_voltage_V | applied voltage in Volt (float), optional |
| _mpif_synthesis_react_electrochem_current_A | applied current in Ampere (float), optional |
| _mpif_synthesis_react_sonication_method | sonication devise (string from {ultrasonic bath, ultrasonic probe, other}), optional |
| _mpif_synthesis_react_sonication_power | sonication power (float), optional |
| _mpif_synthesis_react_sonication_power_unit | unit for _mpif_synthesis_react_sonication_power (string from {W, kHz}), optional |
| _mpif_synthesis_react_photochemical_wavelength_nm | wavelength of the light in nanometer (float), optional |
| _mpif_synthesis_react_photochemical_power_W | power of the light source in Watt (float), optional |
| _mpif_synthesis_react_photochemical_source | light source devise (string), optional |
| _mpif_synthesis_react_temperature_C | reaction temperature in Celsius (float), optional |
| _mpif_synthesis_react_temperature_controller | method of controlling reaction temperature (string from {ambient, oven, oil bath, water bath, dry bath, hot plate, microwave, furnace, other}), optional |
| _mpif_synthesis_react_time | reaction time (float), optional |
| _mpif_synthesis_react_time_unit | unit for _mpif_synthesis_react_time (string from {s, min, h, days}), optional |
| _mpif_synthesis_react_atmosphere | reaction atmosphere (string from {air, dry, inert, vacuum, other}), optional |
| _mpif_synthesis_react_container | reaction container (string), optional |
| _mpif_synthesis_react_note | special note for reaction (string), optional |
| _mpif_synthesis_product_amount | amount of the product (float), optional |
| _mpif_synthesis_product_amount_unit | unit for _mpif_synthesis_product_amount (string from {mg, g, kg, μL, mL, L}), optional |
| _mpif_synthesis_product_yield_percent | product yield in percent (float), optional |
| _mpif_synthesis_scale | scale of the synthesis (string from {milligram, gram, multigram, kilogram}), optional |
| _mpif_synthesis_safety_note | safety note for the synthesis (string), optional |
| Section 4: Synthesis Procedure Details | |
| _mpif_substrate_number | number of substrates (float), **mandatory** |
| _mpif_substrate_id | identification code of the substrate (string: from {R1, R2, …}), **mandatory** |
| _mpif_substrate_name | name of the substrate (string), **mandatory** |
| _mpif_substrate_molarity | molar amount of the substrate (float), optional |
| _mpif_substrate_molarity_unit | unit for _mpif_substrate_molarity (string from {μmol, mmol, mol, kmol}), optional |
| _mpif_substrate_amount | mass or volume of the substrate (float), **mandatory** |
| _mpif_substrate_amount_unit | unit for _mpif_substrate_amount (string from {mg, g, kg, μL, mL, L}), **mandatory** |
| _mpif_substrate_supplier | supplier of the substrate (string), optional |
| _mpif_substrate_purity_percent | purity of the substrate in percent (float), optional |
| _mpif_substrate_cas | CAS registry number of the substrate (string), optional |
| _mpif_substrate_smiles | SMILES of the substrate chemical structure (string), optional |
| _mpif_solvent_number | number of solvents (float), **mandatory** |
| _mpif_solvent_id | identification code of the solvent (string from {S1, S2, …}), **mandatory** |
| _mpif_solvent_name | name of the solvent (string), **mandatory** |
| _mpif_solvent_molarity | molar amount of the solvent (float), optional |
| _mpif_solvent_molarity_unit | unit for _mpif_solvent_molarity (string from {μmol, mmol, mol, kmol}), optional |
| _mpif_solvent_amount | volume of the solvent (float), **mandatory** |
| _mpif_solvent_amount_unit | unit for _mpif_solvent_amount (string from {μL, mL, L}), **mandatory** |
| _mpif_solvent_supplier | supplier of the solvent (string), optional |
| _mpif_solvent_purity_percent | purity of the solvent in percent (float), optional |
| _mpif_solvent_cas | CAS registry number of the solvent (float), optional |
| _mpif_solvent_smiles | SMILES of the solvent chemical structure (string), optional |
| _mpif_vessel_number | number of vessels (float), **mandatory** |
| _mpif_vessel_id | identification code of the vessel (string from {V1, V2, …}), **mandatory** |
| _mpif_vessel_volume | volume of the vessel (float), **mandatory** |
| _mpif_vessel_volume_unit | unit for _mpif_vessel_volume (string from {μL, mL, L}), **mandatory** |
| _mpif_vessel_material | material of the vessel (string), **mandatory** |
| _mpif_vessel_type | type of the vessel (string from {vial, jar, autoclave, beaker, flask, centrifugation tube, other}), **mandatory** |
| _mpif_vessel_supplier | supplier of the vessel (string), optional |
| _mpif_vessel_purpose | purpose of the vessel (string from {storing, reaction, other}), **mandatory** |
| _mpif_vessel_note | special note of the vessel (string), optional |
| _mpif_hardware_number | number of hardware (float), **mandatory** |
| _mpif_hardware_id | identification code of the hardware (string from {H1, H2, …}), **mandatory** |
| _mpif_hardware_purpose | purpose of the hardware (string from {temperature control, atmosphere control, mixing, synthesis devise, transferring, separation, drying, other}), **mandatory** |
| _mpif_hardware_general_name | general name of the hardware (string), **mandatory** |
| _mpif_hardware_product_name | product name of the hardware (string), optional |
| _mpif_hardware_supplier | supplier of the hardware (string), optional |
| _mpif_hardware_note | special note of the hardware (string), optional |
| _mpif_procedure_number | number of procedures (float), **mandatory** |
| _mpif_procedure_id | identification code of the procedure (string from {P1, P2, …}), **mandatory** |
| _mpif_procedure_type | type of the procedure (string from {preparation, reaction, work-up}), **mandatory** |
| _mpif_procedure_atmosphere | atmosphere of the procedure (string from {air, dry, inert, vacuum, other}), **mandatory** |
| _mpif_procedure_detail | detailed note of the procedure (string), **mandatory** |
| _mpif_procedure_full | full details of the procedures (string), optional |
| Section 5: Characterization Information | |
| _mpif_pxrd_data | data name for embedded PXRD data (string), optional |
| _pxrd_source | X-ray source of the PXRD measurement (string from {Cu, Cr, Fe, Co, Mo, Ag, synchrotron, other}), optional |
| _pxrd_lambda | X-ray wavelength in angstrom (float), optional |
| _pxrd_2theta | 2 theta value in degree (float), optional |
| _pxrd_intensity | scattering intensity (float), optional |
| _mpif_tga_data | data name for embedded TGA data (string), optional |
| _tga_temperature_C | TGA temperature in Celsius (float), optional |
| _tga_weight_percent | TGA weight percent (float), optional |
| _mpif_aif | embedded AIF (string), optional |
| _mpif_cif | embedded CIF (string), optional |

1. **Large Language Model Extraction Test**

To evaluate the challenge of extracting synthesis data from scientific literature, a benchmark test was designed to assess the performance of state-of-the-art large language models (LLMs).

Four LLMs were tested: Gemini 2.5 Pro, Gemini 2.5 Flash, Claude 3 Sonnet and Claude 3.1 Opus. The models were tasked with extracting information for three different metal-organic frameworks (MOFs): PCN-222,^[1]^ JUK-8-ip,^[2]^ and WaaF-1.^[3]^ The source texts provided to the models were the full pdf research articles (including main text and supporting information).

Each LLM received a standardized prompt instructing it to extract ten specific data fields, including reaction temperature, time, and yield. The prompt contained strict rules for the LLMs to follow:

```

Extract the following fields for the material *(material identification)* from the given text. Only use explicitly stated information — do not infer or assume any values.

Fields to extract:

- _mpif_product_type: Type of product (string from {MOF, COF, composite, other})

- _mpif_product_name_common: common name of the product (string)

- _mpif_product_formula: chemical formula of the product (string or null)

- _mpif_synthesis_type: type of the synthetic procedure (string from {mix, diffusion, evaporation, microwave, mechanochemical, electrochemical, sonochemical, photochemical, flow, other})

- _mpif_synthesis_react_temperature_C: reation temperature in celcius (float)

- _mpif_synthesis_react_time: reaction time (float)

- _mpif_synthesis_react_time_unit: unit for _mpif_synthesis_react_time (string from {s, min, h, days})

- _mpif_synthesis_product_yield_percent: product yield in percent (float)

- _mpif_audit_contact_author_name: name of the responsible author (string)

_ _mpif_audit_contact_author_address: business address of the responsible author (string)

Output Format:

Return a valid JSON object with the exact field names above. Use `null` for any field not explicitly mentioned in the text.

Example Output:

{

"_mpif_product_type": "MOF",

"_mpif_product_name_common": "AMOF-1",

"_mpif_product_formula": "Cu2C6H4",

"_mpif_synthesis_type": "mix",

"_mpif_synthesis_react_temperature_C": 70,

"_mpif_synthesis_react_time": 24,

"_mpif_synthesis_react_time_unit": "h"

"_mpif_synthesis_product_yield_percent": 42,

"_mpif_audit_contact_author_name": "Morgan Collins",

"_mpif_audit_contact_author_address": "University ABC, 123 Fake Street, 1000 Australia"

}

Important Rules:

1. DO NOT infer or guess any field. Only extract if explicitly stated.

2. Preserve multi-line notes using '\n' as newline separator.

3. Return ONLY the JSON object — no additional text or explanation.

```

The JSON output from each LLM was then programmatically compared to a manually created "ground truth" JSON file for each material generated from the MPIF. Finally, these results were aggregated across all tests to calculate the overall percentage of correctly identified values for each field (Figure 1), revealing the significant difficulty LLMs have in reliably parsing unstructured scientific text.


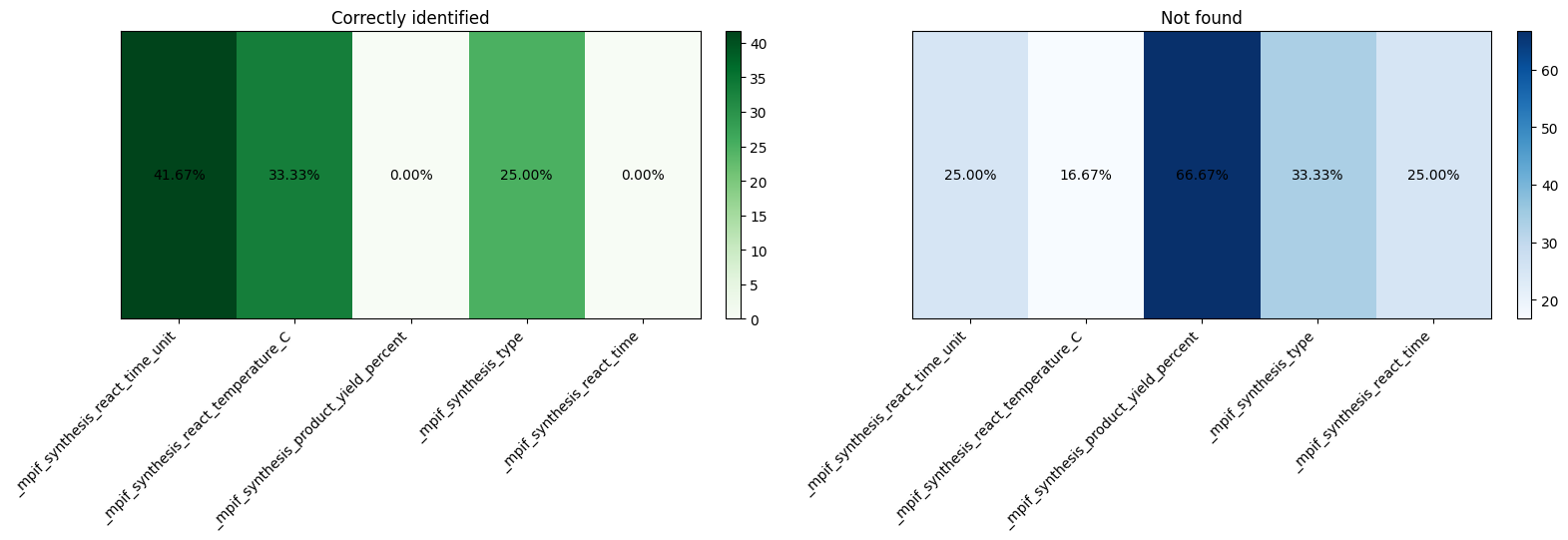


**Figure S1.** Summary of the average error and not found rates for LLMs extracting the specified metadata from the published texts.

1. **References**

[1] D. Feng, Z.-Y. Gu, J.-R. Li, H.-L. Jiang, Z. Wei, H.-C. Zhou, “Zirconium-Metalloporphyrin PCN-222: Mesoporous Metal–Organic Frameworks with Ultrahigh Stability as Biomimetic Catalysts” *Angewandte Chemie International Edition* **2012**, *51*, 10307–10310.

[2] K. Roztocki, F. Formalik, A. Krawczuk, I. Senkovska, B. Kuchta, S. Kaskel, D. Matoga, “Collective Breathing in an Eightfold Interpenetrated Metal–Organic Framework: From Mechanistic Understanding towards Threshold Sensing Architectures” *Angewandte Chemie International Edition* **2020**, *59*, 4491–4497.

[3] S. Tokuda, S. Furukawa, “Three-dimensional van der Waals open frameworks” *Nat. Chem.* **2025**, *17*, 672–678.
